# Development of a Zr-Based Metal-Organic Framework (UiO-66) for a Cooperative Flame Retardant in the PC/ABS

**DOI:** 10.3390/polym16142083

**Published:** 2024-07-21

**Authors:** Shaojun Chen, Zerui Chen, Weifeng Bi, Wei Du, Ling Lin, Dasong Hu, Haitao Zhuo

**Affiliations:** 1 College of Materials Science and Engineering, Shenzhen University, Shenzhen 518053, China; chensj@szu.edu.cn (S.C.); czr1079975344@163.com (Z.C.); a773122527@163.com (W.B.); 15368563584@163.com (W.D.); 2023200132@email.szu.edu.cn (L.L.); 2021200135@email.szu.edu.cn (D.H.); 2 College of Chemistry and Environment Engineering, Shenzhen University, Shenzhen 518053, China

**Keywords:** metal-organic framework, flame retardance, cooperative effect, PC/ABS

## Abstract

Polycarbonate/acrylonitrile butadiene styrene (PC/ABS) blends are widely used as engineering plastic alloys; however, they have a low fire safety level. To improve the flame-retardant property of PC/ABS, a zirconium-based metal-organic framework material (UiO-66) was synthesized with zirconium chloride and terephthalic acid and used as a flame-retardant cooperative agent. Its flame-retardant performance and mode of action in the PC/ABS blends were carefully investigated. The results showed that UiO-66 had good thermal stability and delayed the pyrolysis of the materials, thus significantly enhancing the efficiency of intumescent flame retardants. By compounding 7.0 wt% hexaphenyloxy-cyclotri-phosphazene (HPCTP) with 3.0 wt% UiO-66, the PC/ABS blends reached a limiting oxygen index value of 27.0% and V0 rating in the UL-94 test, showing significantly improved resistance to combustion dripping. In addition, UiO-66 enhanced the smoke and heat suppression characteristics of the intumescent flame-retardant materials. Finally, the flame-retardant mode of action in the blends was indicative of UiO-66 having a cooperative effect on the flame-retardant performance of PC/ABS/HPCTP materials. This work provides good ideas for further development of the flame-retardant ABS/PC.

## 1. Introduction

Polycarbonate (PC)/acrylonitrile butadiene styrene (ABS) is a typical polymer blend made of PC and ABS resin. PC confers strength and a high heat-deflection temperature, whereas ABS possesses an easy processing ability and is cheap [1,2]. Thus, PC/ABS has become one of the most widely used plastics in the electrical and electronics industries [3]. However, it is easily ignited in the air. To expand its flame retardancy application in automotive parts, 5G communication equipment, and household appliances, it is, therefore, necessary to improve the flame-retardant properties of PC/ABS. Various kinds of flame retardants have been used in the PC/ABS. For example, phosphazene compounds are known for releasing less smoke and having a lower toxicity than other phosphorus compounds when burning, making them environmentally friendly flame retardants [4]. Particularly, hexaphenyloxy-cyclotri-phosphazene (HPCTP) provides flame-retardant properties through condensed and partial gas-phase mechanisms. For example, the phosphonitrile-triazine bis-alkyl flame retardant (A3) with an aniline end-group could form a more complete and dense char layer when PC burnt and released PO· to act as a flame retardant in the gas phase [5]. It was also reported that the aromatic polyimide (API) charging agent could fill in the toughness gap of HPCTP and then enhance the flame-retardant properties of PC, acting as a co-efficient flame retardant to improve notched impact properties [6]. During combustion, HPCTP decomposes into phosphoric acid, polyphosphoric acid, and N_2_ non-flammable gases. This promotes the dehydration of PC/ABS into a carbonaceous char that expands to form an intumescent char layer, thus inhibiting heat transfer and oxygen access [7]. However, many previous studies have shown that HPCTP has a relatively low flame-retardant efficiency, requiring the addition of 15 parts of cooperative agents to achieve a UL94 V0 rating [4,8]. Therefore, there is an urgent need to develop a new flame-retardant cooperative agent to modify the intumescent flame-retardant properties of HPCTP.

Metal-organic frameworks (MOFs) are a class of porous crystalline materials formed via the coordination between metal ions or clusters and organic ligands. Compared to traditional inorganic porous materials, MOFs have distinct advantages such as high porosity, tunable pore sizes and topologies, organic–inorganic hybrid properties, and the natural coexistence of metallic and heteroatoms. Thus, they have a wide range of applications, such as for smart sensors [9], gas storage [10], CO_2_ fixation [11], and are commonly referred to as “versatile materials”. For example, carboxyl-substituted porphyrin derivatives were successfully synthesized via MOFs materials (UiO-66 and UiO-67) [12], and they contributed to the development of porphyrin-based functional materials. In recent years, MOFs have gained increasing attention as emerging flame retardants. Owing to their natural porous structure, tunable and modifiable characteristics, and rich metallic components, MOFs are beneficial for enhancing the fire safety performance of polymers. In 2008, researchers reported the successful and reproducible synthesis of a new zirconium-based MOF named UiO-66, which comprises zirconium clusters and organic ligands with the molecular formula [Zr_6_O_4_(OH)_4_L_6_]_n_, where L represents linear dicarboxylic acid ligand [13]. However, scholars have found broad diffraction peaks in XRD patterns, indicating low crystallinity [14,15]. It implied that the synthesis of UiO-66 could be regulated with acetic acid to improve its regularity and particle size [16], thereby significantly enhancing the success rate of synthesis. In 2019, zirconium-based MOFs (Zr-BDC) were used to improve the fire safety performance of PC, increasing the time to ignition (TTI) and reducing fire hazards [17]. The addition of 4 wt% Zr-BDC allowed PC to achieve a UL-94 V0 rating, conferring PC with a better char-forming ability (catalytic charring) and smoke suppression property during pyrolysis, which are necessary for personnel evacuation and rescue measures. However, there are only a few reports on their application in PC/ABS blends. 

In this work, a flame retardant with zirconium-based metal-organic frameworks (MOFs) was synthesized to enhance the flame-retardant properties of PC/ABS. First, MOFs (UiO-66) was synthesized by Zr_6_O_4_(OH)_4_ clusters and 1,4-benzenedicarboxylate ligands. Then, a small amount of UiO-66 combined with HPCTP was melt-blended with PC/ABS to produce the intumescent flame-retardant material, termed UiO-66@HPCTP@PC/ABS. Finally, the structures of the UiO-66 samples was characterized, and their flame-retardant properties and flame-retardant mode of action in the blends were studied carefully. This work presents a highly efficient flame-retardant cooperative agent for the development of high-performance flame-retardant plastics.

## 2. Experimental Section

### 2.1. Materials

PC (injection grade, QiMei Company, Taiwan), ABS (injection grade, QiMei Company, Taiwan, China), zirconium chloride (98% ZrCl_4_, Shanghai Aladdin Biochemical Company, Shanghai, China), terephthalic acid (99% TPA, Shandong Yusuo Chemical Technology Co., Ltd., Heze, China), hexaphenyloxy-cyclotri-phosphazene (99% HPCTP, Jinan Sino New Material Technology Co., Ltd., Heze, China), N,N-dimethylformamide (99.5% DMF, Shanghai Aladdin Biochemical Company, Shanghai, China), ethanol (99% EtOH, Shanghai Aladdin Biochemical Company, Shanghai, China), and acetic acid (99.8% EtOH, Shanghai Aladdin Biochemical Company, Shanghai, China) were used without further purification for the experiments.

### 2.2. Synthesis of UiO-66

**Figure 1:**
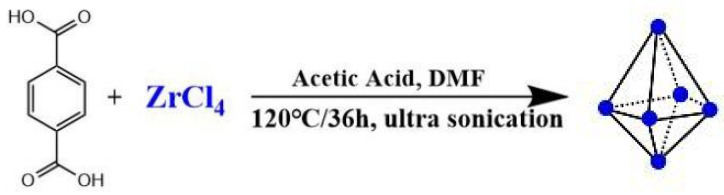
Synthetic route of UiO-66.

The synthetic route of UiO-66 is presented in Figure 1, and the 3D/2D structure is shown in Figure S1. First, 1.98 g ZrCl_4_ and 1.76 g TPA were dissolved in 150 mL anhydrous DMF, followed by sonication for 20 min. Then, 4 mL of acetic acid was added, followed by sonication for 10 min. The solution was transferred to a blue bottle and placed into a 120 °C electric blast drying chamber. After solvent removal for 36 h, the powder was cooled down to 25 °C, centrifuged at 6000 rpm for 15 min to filter the white precipitate, and washed with DMF and ethanol three times. Finally, UiO-66 was obtained after drying in an 85 °C constant-temperature vacuum drying oven for 36 h.

### 2.3. Synthesis of UiO-66@HPCTP@PC/ABS

The PC/ABS flame-retardant materials (UiO-66@HPCTP@PC/ABS) were prepared by melt blending and a twin-screw extrusion. The components and ratios of samples are shown in Table 1. First, PC and ABS resins were weighed and mixed (70:30) and dried in a 70 °C electric heating blast drying oven for 4 h. Then, specified amounts of HPCTP and UiO-66 were added to PC and ABS, according to Table 1. After all components were mixed evenly, the materials were added to a twin-screw extruder. The temperature of each zone in the twin-screw extruder was set at 210, 220, 230, 240, 250, 250, 250, 245, and 245 °C, and the screw speed was set to 15~20 rpm. After melt blending, pellets were prepared in the pelleting machine and further dried in an electric blast drying box at 70 °C for 4 h. The test specimens were injected according to the GB/T 17037.1-1997 standard or GB/T 9341-2008 [18,19]. The injection temperature was 230 °C, and the injection pressure was 90 MPa.

### 2.4. Characterizations

FT-IR spectroscopy was conducted on a Nicolet 6700 infrared spectrometer (Thermo Fisher Scientific, Waltham, MA, USA), with a scanning speed of 4 cm^−1^ and scanning range of 4000–500 cm^−1^. The attenuated total reflection (ATR) mode and the KBr pressurization method was used to measure the samples.

X-ray photoelectron spectroscopy (XPS) was conducted on a Kratos Axis Ultra DLD XPS instrument from Shimadzu Corporation. The powder was cut into a 5 mm × 4 mm sample. The chemical states of C1s, Zr 3d, P 2p, and O1s, among others, were determined by scanning the sample surface.

SEM was conducted using a scanning electron microscope (NGB 4-DXS-10AC, Nanjing Grand Technology Co., Ltd., Nanjing, China). The sample was broken in liquid nitrogen and was adhered to the powder sample.

Differential scanning calorimetry (DSC) curves were recorded using a DSC 25 cell (TA Instrument, New Castle, DE, USA) with a nitrogen protection of 40 mL/min. Approximately 5–10 mg was dried in an 85 °C electric thermostatic drying oven for 2 h and pressed into an aluminum crucible tablet. 

The TG and DTG curves were obtained using the TGA 55 (TA Instrument, New Castle, DE, USA) at a flow rate of 50 mL/min in a nitrogen atmosphere. The heating rate was 10 °C/min, and the temperature range varied from room temperature to 800 °C.

A vertical burning test was performed to measure flame-retardant performance. Each sample was analyzed three times, and the average of the three readings was used to determine its corresponding flame-retardant level.

The limiting oxygen index (LOI) was determined using a JF-3 oxygen index instrument (Shenzhen, China) according to standard GB/T 2406.2-2009 [20], with specimen dimensions of 150 mm × 4 mm × 10 mm.

The CCT was conducted using a British FTT cone calorimeter, according to ISO5660-1 and ASTM D7309 standards [21,22], with an irradiance power of 50 kW/m^2^ and sample dimensions of 100 mm × 100 mm × 3 mm.

The fire growth index (FGI) was used to evaluate the fire safety performance of materials. FGI was calculated as the ratio of PHRR to HRR, with a lower value indicating that the material takes less time to reach a state of intense combustion, thus presenting a lower fire hazard. 

Raman spectroscopy was performed using a Laser Confocal Microscope Raman Spectrometer (INVIA, RENISHAW, London, England). The excitation source was a 514.5 nm argon ion laser. The experiments were performed at 25 °C and tested at a scanning range of 500~2500 cm^−1^.

TG-IR was performed on an FT-IR spectrometer (SPECTRUM TWO, PerkinElmer, MA, USA) equipped with a TGA analyzer (TGA 4000, PerkinElmer, MA, USA) through a heat transfer line, allowing for in situ characterization of decomposition products with a spectral resolution of 4 cm^−1^ and a scanning interval of 2.23 s.

XRD spectroscopy was performed on an X-ray diffractometer (Miniflex600, Rigaku, Tokyo, Japan), with a scanning speed of 10°/min and a scanning range of 5° to 70°.

## 3. Results and Discussion

### 3.1. Preparation of UiO-66

**Figure 2:**
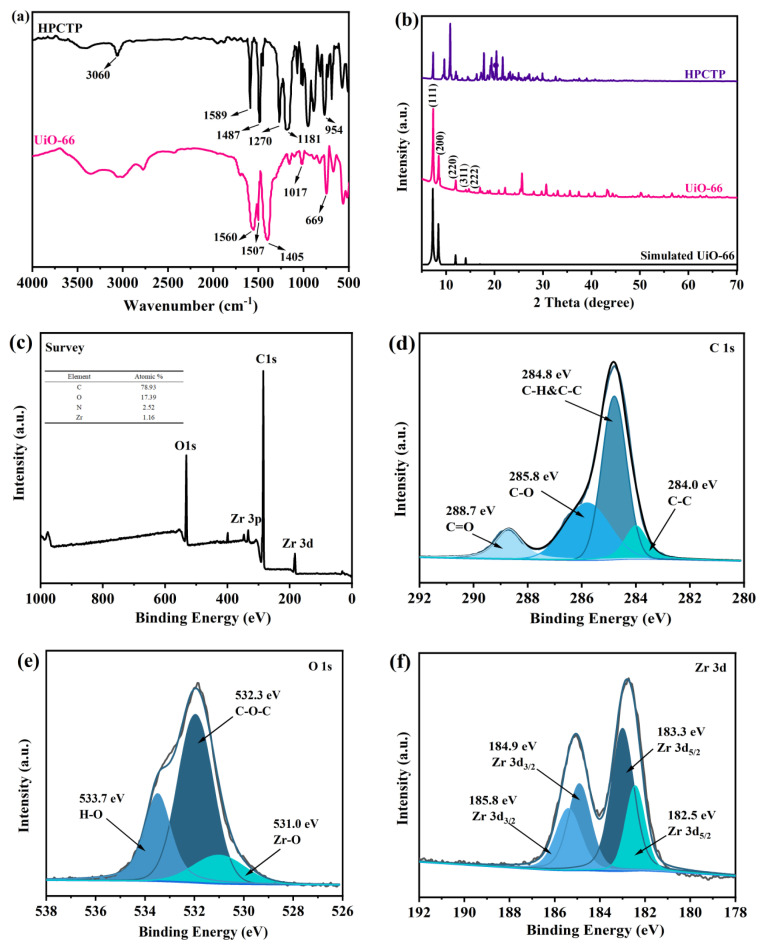
Structure analysis: (**a**) FT−IR spectra of UiO-66 and HPCTP, (**b**) XRD patterns and simulations of UiO-66 and HPCTP, (**c**) XPS survey spectra of UiO-66, (**d**) XPS spectra of C 1s, (**e**) XPS spectra of O 1s, and (**f**) XPS spectra of Zr 3d.

Infrared spectroscopy was used to characterize the synthesized UiO-66 and the selected flame retardant of HPCTP. As shown in the FT-IR spectra in Figure 2a, the asymmetric stretching vibration of Zr-(OC) appears at 669 cm^−1^. The absorption peaks at 1017 cm^−1^ and 1507 cm^−1^ confirm the coordination of terephthalic acid ligands with Zr. The peaks near 1405 cm^−1^ and 1560 cm^−1^ belong to the symmetric and asymmetric vibration peaks of O-C-O in terephthalic acid ligands, respectively. The absorption at the range of 3200~3600 cm^−1^ features the characteristic peak of an O-H stretching vibration, indicating the successful synthesis of UiO-66 [23]. In the FT-IR spectrum of HPCTP, the characteristic peak for the stretching vibration of unsaturated =C-H in the benzene ring is observed at 3060 cm^−1^. The peaks at 1589 cm^−1^ and 1487 cm^−1^ belong to the characteristic peaks of the benzene ring’s skeletal vibration, indicating the presence of a benzene ring structure. The peaks at 1270 cm^−1^ and 1181 cm^−1^ belong to the P=N stretching vibration of cyclic triphosphazene, and the characteristic absorption peak at 954 cm^−1^ represents P-O-C. These results are consistent with previous reports [24].

In the XRD spectra, the main diffraction peaks of UiO-66 are essentially consistent with those of simulated UiO-66. There are distinct sharp peaks at 7.38°, 8.52°, 12.06°, 14.15°, and 14.78°, corresponding to the (111), (200), (220), (311), and (222) crystal planes, respectively. These results confirm the successful synthesis of UiO-66 [25]. Moreover, HPCTP has a high degree of crystallinity and good molecular symmetry, with many sharp diffraction peaks in its spectrum. The positions of peaks at 7.2°, 9.4°, 10.7°, 17.5°, and 20.4° match those previously reports [7].

XPS was performed to detect the types of elements and bonding in UiO-66. From the XPS survey spectra, C 1s, O 1s, and Zr 3d XPS bands were detected. The C 1s peaks were distributed across four groups of peaks at 284.0 eV, 284.8 eV, 285.8 eV, and 288.7 eV. The signals at 284.0 eV and 284.8 eV corresponded to C-C bonds, while those at 285.8 eV and 288.7 eV corresponded to C-O and C=O or the connection between the benzene ring and carboxyl groups. In addition, the O 1s spectra can be fitted to three peaks. The peak near 532.3 eV indicates that C-O-C at 533.7 eV is attributed to H_2_O, and that at a peak at 531.0 eV corresponds to Zr-O bonds. The Zr 3d has four peaks: the signals at 184.9 eV and 185.8 eV belong to Zr 3d3/2, and those at 183.3 eV and 182.5 eV correspond to Zr 3d5/2. Therefore, these results confirm the successful synthesis of UiO-66.

**Figure 3:**
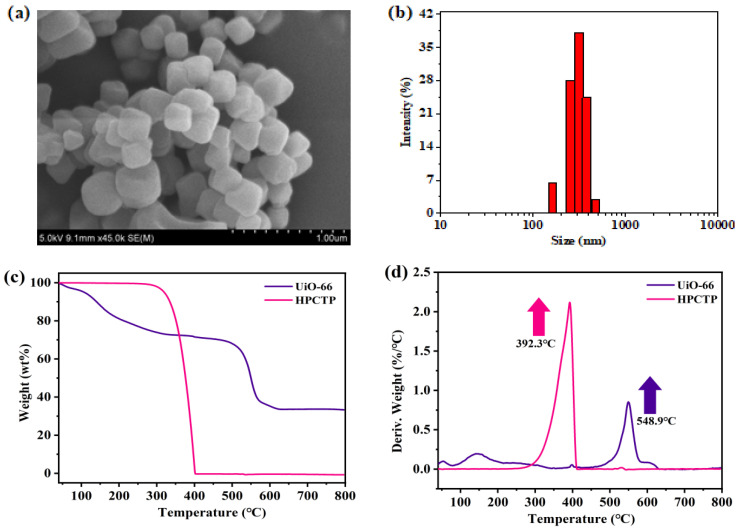
Morphology ((**a**) SEM, (**b**) particle size distribution) of UiO-66 and thermal-stability ((**c**) TG curves, (**d**) DTG curves) of UiO-66 and HPCTP.

SEM images show that the UiO-66 nanocrystals have dispersed octahedral morphology with a uniform crystal size (Figure 3). The average size of the nanocrystals is approximately 350 nm. EDS shows that the elements zirconium and oxygen are evenly distributed throughout the materials (Figure S1). TGA is performed to analyze the thermal stability of HPCTP and UiO-66. UiO-66 has a slight weight loss peak near 144.8 °C, which is attributed to the desorption of DMF and ethanol. At the temperature range of 200~500 °C, there is a minor weight loss of UiO-66 that may be attributed to the removal of hydroxyl groups from the zirconium clusters to form water. Above 500 °C, the MOF structure begins to collapse and pyrolyze, with the maximum thermal weight loss rate temperature (T_MAX2_) reaching approximately 548.9 °C, indicating that the obtained UiO-66 has high thermal stability with a residual mass rate of 33.92 wt% at 800 °C. Additionally, the pure HPCTP has only one T_MAX_ (392.3 °C), and its mass rapidly decreases as the temperature is increased, with an approximate residual rate of 0 wt% at 400 °C (Table S1). These findings imply that HPCTP by itself does not possess good thermal stability and needs to be mixed with UiO-66 to achieve good flame retardancy.

### 3.2. Preparation of UiO-66@HPCTP@PC/ABS

UiO-66 and HPCTP were both used to modify the flame retardancy of PC/ABS. FT-IR spectroscopy was performed to characterize the structure of PC/ABS and the flame-retardant samples, e.g., HP-7, U-3, and HPU-3. The stretching vibration peaks of aromatic and aliphatic C-H in the PC/ABS were detected at 3200~3000 cm^−1^ and 3000~2800 cm^−1^, respectively. The peaks at 2237 cm^−1^ and 911 cm^−1^ correspond to the nitrile C≡N of ABS and the C-H bond connected to PB, respectively. Those peaks at 1,772 cm^−1^, 1495 cm^−1^, and 1200 cm^−1^ belong to the carbonyl stretching vibration, aromatic ring stretching vibration, and C-O stretching vibration in PC, respectively, representing the main groups of PC/ABS. After adding HPCTP, a weak absorption peak near 949 cm^−1^ was detected in HP-7, corresponding to P-O-C stretching. The U-3 sample showed a new absorption peak at 1,405 cm^−1^, which was attributed to the symmetric vibration peak of O-C-O from the terephthalic acid ligands of UiO-66. The HPU-3 sample displayed both P-O-C and O-C-O characteristic peaks. These results indicate that the UiO-66@HPCTP@PC/ABS blends were successfully prepared.

**Figure 4:**
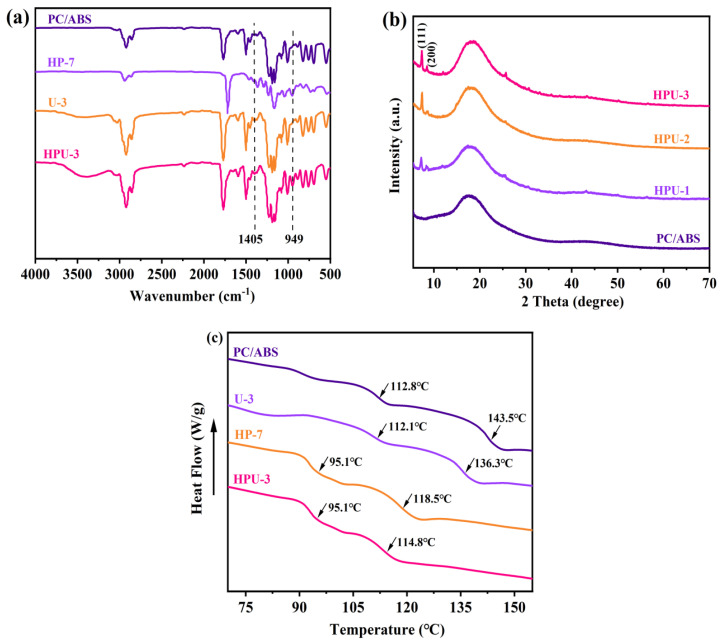
(**a**) FT-IR spectra of PC/ABS, HP-7, U-3, and HPU-3 samples. (**b**) XRD spectra of PC/ABS and HPU-n series. (**c**) DSC curves of PC/ABS, HP-7, U-3, and HPU-3 samples.

Figure 4b shows a broad and diffuse PC/ABS diffraction peak. With an increase in the amount of UiO-66, characteristic sharp peaks at 7.38 ° and 8.52 ° appeared, corresponding to the (111) and (200) planes of UiO-66, respectively. Thus, UiO-66 was successfully introduced to PC/ABS.

DSC was performed to characterize the phase structure and transition behaviors of UiO-66 and its blends. As shown in Figure 4c, the incorporation of UiO-66 led to a slight reduction in the Tg of the PC and SAN phases in ABS, indicating that UiO-66 particles might be incorporated to the molecular chains of resins to serve as lubricants, which increases free volume and enhances segmental mobility. Moreover, HPCTP significantly reduces the Tg of the aforementioned two phases. The benzene ring structure in HPCTP interacts with the SAN and PC phases through π-π stacking to form weak hydrogen bonds [26], plasticizing the PC/ABS system containing benzene ring structures. Therefore, with the addition of HPCTP and UiO-66, the flame-retardant material, PC/ABS, is endowed with better processing performance.

**Figure 5:**
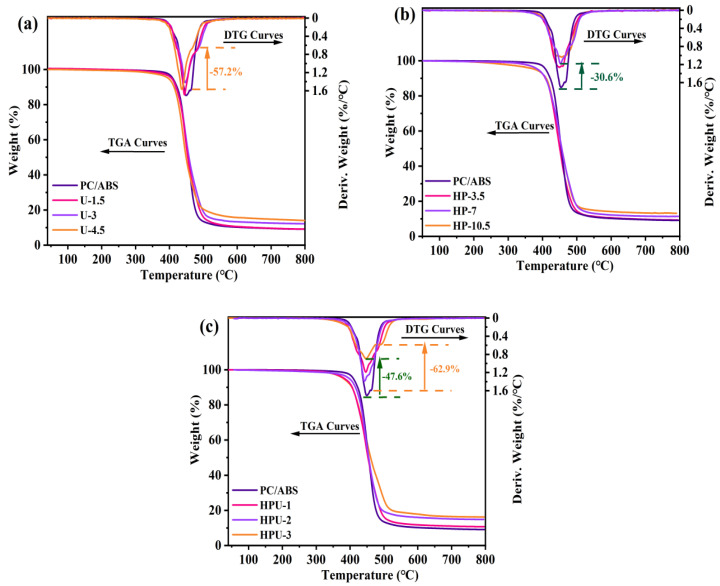
TGA and DTG curves of the PC/ABS blends: (**a**) UiO-66 flame-retardant series sample (U), (**b**) intumescent flame-retardant system sample (HP), and (**c**) UiO-66 cooperative intumescent flame-retardant system sample (HPU).

Figure 5 presents the TGA and DTG curves of the HPCTP intumescent, UiO-66, and the UiO-66 cooperative intumescent flame-retardant series. Pure PC/ABS has a two-step degradation process—matrix thermal degradation (410~500 °C) and deep cracking, such as chain cyclization and crosslinking (540~630 °C). However, after introducing UiO-66, the main degradation process of UiO-66 occurs between the two degradation stages of PC/ABS, implying that UiO-66 may play a role throughout the thermal degradation process of the material.

Figure 5a shows that the U-3 sample’s DTG shoulder peak was reduced by 57.2% at 490 °C and that char yield was significantly increased at 500 °C and 800 °C (Table S2), suggesting that UiO-66 had a significant delay effect on the thermal decomposition of PC/ABS. Additionally, samples U-1, 2, and 3 tended to degrade slightly at a low temperature range, possibly due to minor PC/ABS chain scission in the early stages of TGA. As shown in Figure 5b, compared to the pure PC/ABS, sample HP-7 has a maximum weight loss rate of 30.6%. This implies that the intumescent flame-retardant material promotes the dehydration of combustion products into char to form an expanded char layer, thereby improving the thermal stability. Figure 5c displays the TGA and DTG curves of UiO-66 cooperative intumescent flame-retardant materials. The HPU-3 sample shows a 47.6% decrease in the maximum thermal weight loss rate and a 62.9% reduction in the DTG shoulder peak. The temperature of the DTG shoulder peak increases from 463.2 °C to 493 °C. The char yield at 800 °C increases from 9.0 wt% to 16.4 wt% (Table S2). These results imply that UiO-66 has a significant cooperative flame-retardant effect and causes a noticeable delay in the thermal decomposition process. The increase in char yield suggests that zirconium and organic ligands may participate in the carbonization process during thermal degradation.

### 3.3. Performances of Flame Retardant

Vertical burning and LOI tests are two common methods used to characterize the flame retardancy of polymers. Table 2 summarizes the results of vertical burning tests for materials containing different amounts of UiO-66 and HPCTP. Samples HP-3.5, HP-7, and HP-10.5 exhibited dripping phenomena during combustion, but only HP-10.5 containing 10.5 wt% HPCTP achieved a UL-94 V-2 rating. However, as engineering plastics, they still did not meet the requirements for fire safety standards.

The pure PC/ABS has an LOI value of 21.2% and shows noticeable melt dripping that can ignite cotton during combustion. After adding UiO-66, particularly in sample U-4.5, this melt dripping phenomenon was effectively suppressed, and its LOI value increased to 24.1%. Since the preparation of UiO-66 is rather costly and intricate owing to complex procedures, it is rational to combine it with intumescent flame-retardant systems. From Table 1, by compounding 7.0 wt% HPCTP with 1, 2, and 3 wt%, UiO-66, HPU-1, HPU-2, and HPU-3 had increased LOI values of 23.8%, 25.2%, and 27.0%, respectively. Among them, only HPU-3 reached a V-0 rating, showing significantly improved resistance to combustion dripping. Therefore, UiO-66 alone cannot significantly enhance the efficiency of intumescent flame retardants in HPCTP.

**Figure 6:**
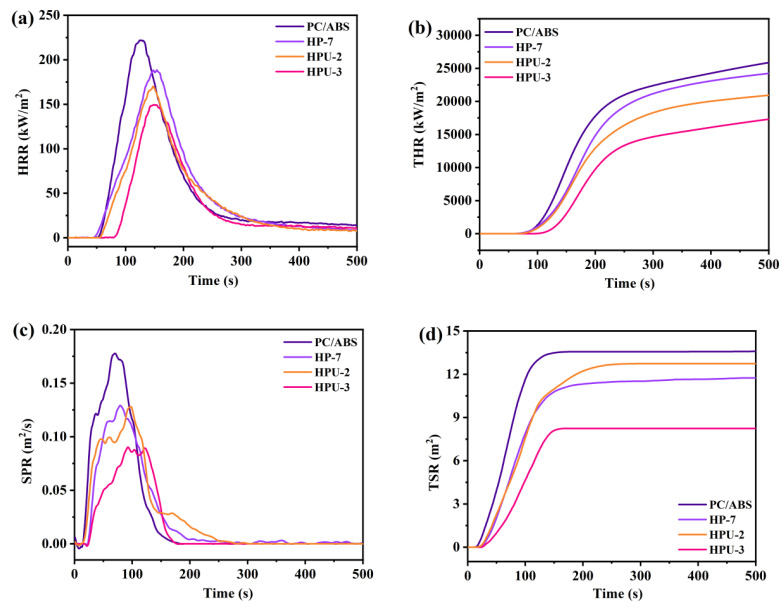
(**a**) Heat release rate (HPR), (**b**) total heat release (THR), (**c**) smoke production rate (SPR), and (**d**) total smoke production (TSR) curves of the PC/ABS blends.

At fire scenes, heat radiation or toxic smoke produced during combustion leads to many casualties [27]. Currently, the CCT is widely used to simulate the fire behavior of polymeric materials in real fire scenarios [28] and has two main parameters: heat release rate (HRR) and total heat release (TRR). As shown in Figure 6, compared to those of PC/ABS, the peak HRR (PHRR) and TRR of sample HP-7 decrease by 15.1% and 6.3%, respectively, while those of sample HPU-3 decrease by 32.2% and 33.1% after introducing UiO-66 (Table S3). This confirms that UiO-66 enhances the suppression of heat release in the intumescent flame-retardant system. In the initial stage of combustion, HPU-3 releases heat more slowly, as the MOF does not decompose in the early stages of pyrolysis, acting as a physical flame retardant. Furthermore, MOFs can provide smoke suppression effects [29]. As shown in Figure 6c,d, compared to that of the pure PC/ABS and sample HP-7, the smoke production rate (SPR) of HPU-3 significantly decreases as the peak SPR (TSPR) and total smoke release (TSR) are reduced by 39.3% and 49.4%, respectively (Table S3). From Figure 3d, the UiO-66 framework structure begins to collapse at approximately 450 °C, requiring higher temperatures for complete collapse. Although the internal temperature of polymeric materials can reach 700 °C or even higher during combustion, the framework structure remains relatively intact in the early stages of pyrolysis. When smoke is produced, most pyrolysis gases need to overcome the tunneling effect, traveling through the labyrinthine interior of the crystals, thereby delaying the smoke release in the early stages of pyrolysis. Additionally, UiO-66, which contains metals, metal oxides, and ligands with benzene ring structures, participates in the matrix carbonization reaction, making the char more compact, thus enhancing the smoke and heat suppression characteristics of the intumescent flame-retardant materials.

**Figure 7:**
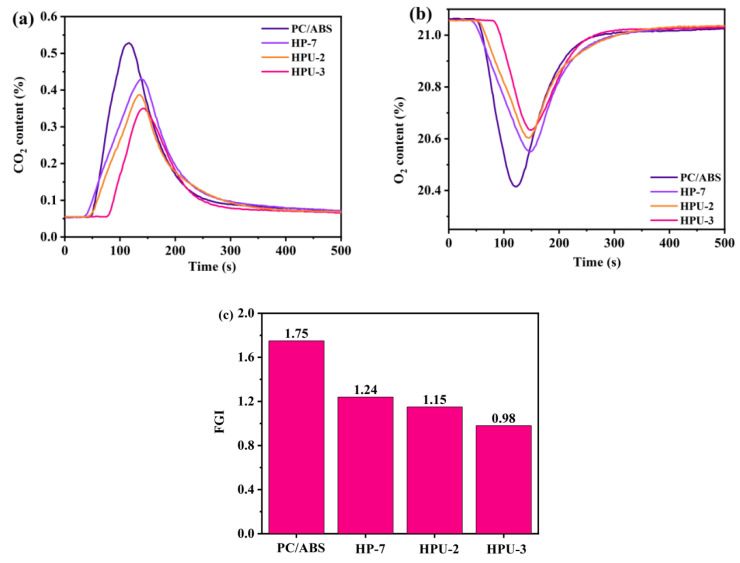
Changes in CO_2_ (**a**) and O_2_ contents (**b**) in relation to the time and (**c**) fire growth index (FGI) of PC/ABS blends.

Figure 7 illustrates the changes in CO_2_ and O_2_ contents during CCT. Upon the addition of UiO-66, the O_2_ peak gradually increases, whereas that of CO_2_ decreases. This result indicates that the flame-retardant system reduces the intensity of PC/ABS combustion by inhibiting the contact between the matrix and O_2_, thereby diminishing its reaction with O_2_. Figure 7c shows that the FGI value of HP-7 containing only HPCTP is 1.24, whereas those of HPU-2 and HPU-3 containing both UiO-66 and HPCTP decrease to 1.15 and 0.98, showing 7.3% and 21.0% reductions, respectively. This result confirms that UiO-66 significantly improves fire safety performance.

### 3.4. Flame-Retardant Mode of Action

**Figure 8:**
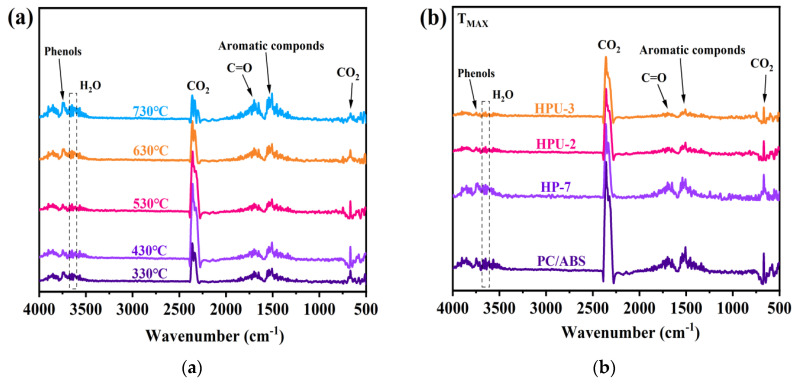
TG-IR spectra: (**a**) PC/ABS at different temperatures; (**b**) PC/ABS, HP-7, HPU-1, HPU-2 and HPU-3 at maximum weight loss temperature (T_MAX_).

The flame-retardant mode of action was investigated by analyzing the composition of pyrolysis gases and residues from vertical burning tests. TG-IR was used to identify the composition of pyrolysis gases from PC/ABS at different temperatures. As shown in Figure 8, the absorption peaks at 2349 cm^−1^ and 667 cm^−1^ represent CO_2_ stretching and bending vibrations, respectively. The region between 3750~3735 cm^−1^ corresponds to phenol and its derivatives, while that between 3720~3600 cm^−1^ corresponds to water vapor. The peak at 1780 cm^−1^ represents ester oligomers, and that at 1500 cm^−1^ is associated with aromatic compounds. Minor ether features are detected between 1285~1120 cm^−1^. Figure 8b shows the infrared spectrum measured at T_MAX_, where 7 wt% HPCTP has reduced the volatilization concentration of CO_2_ from pure PC/ABS. Characteristic peaks for phosphorus-containing organics are not found, which may be attributed to their release in amounts that are too small to be detectable. At T_MAX_, the absorption peaks for phenolic derivatives, ester compounds, and other hydrocarbons, such as aromatics, in HPU-2 and HPU-3 are significantly lower than those in HP-7 and pure PC/ABS. This result suggests that UiO-66 affects the thermal decomposition process of this material as a flame retardant in the solid phase. This might have resulted from the formation of a new compound with a more cross-linked and higher-molecular-weight network structure, reducing the release of organic volatiles.

**Figure 9:**
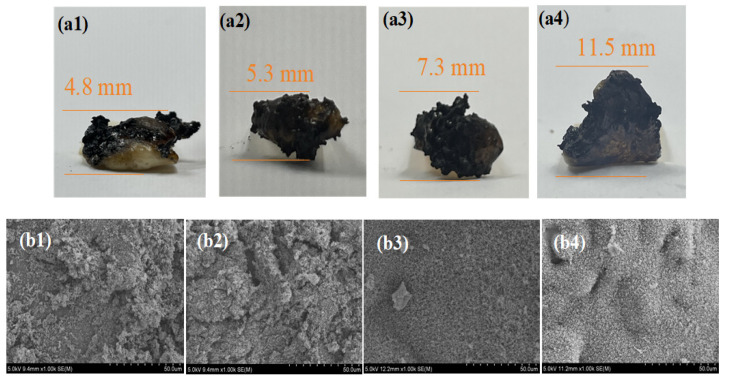
Micromorphology and SEM images of carbon residue after vertical combustion test: (**a1**,**b1**) PC/ABS, (**a2**,**b2**) HP-7, (**a3**,**b3**) HPU-2, and (**a4**,**b4**) HPU-3.

The char residue formed during combustion affects the release of heat and smoke. The carbonization process in burning is often closely related to the amount of residual char, macro-morphology, and micro-structure. Therefore, it is necessary to observe the morphology of charred residues. As shown in Figure 9a, pure PC/ABS has the lowest char formation height, with more products dispersing into the atmosphere as smoke during combustion. In contrast, HPU-3 has a higher expansion degree, continuity, and integrity. Figure 9b presents the corresponding SEM images of the char layers for each sample. The char layer of pure PC/ABS shows many pores and low density owing to the extensive escape of various aromatic derivatives, CO_2_, H_2_O, and phenolic compounds during pyrolysis. The penetration of O_2_ through the loose and porous char layer further exacerbates the burning process on the substrate surface. Although the density of the char layer of HP-7 increases, many pores remains present. Sample HPU-3 has a reduced pore size and a dense and smooth surface char layer. Hence, it is confirmed that UiO-66 enhances the char-forming process of the intumescent flame-retardant material during combustion.

**Figure 10:**
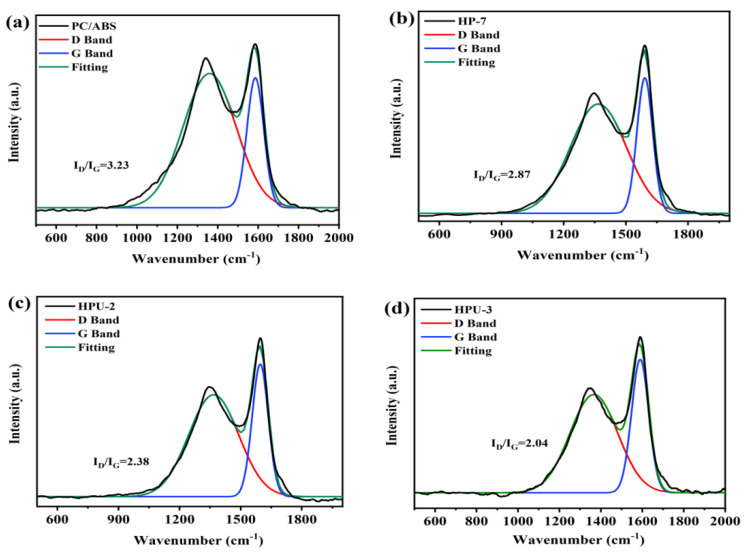
Raman spectra of PC/ABS (**a**), HP-7 (**b**), HPU-2 (**c**), and HPU-3 (**d**) carbon residues.

Raman spectroscopy was used to characterize the degree of graphitization of the char layer. The D peak near 1,350 cm^−1^ and G peak near 1,600 cm^−1^ correspond to amorphous and graphitic carbons, respectively. A higher degree of graphitization results in better barrier effects against heat and oxygen. The integral ratio of the D peak to the G peak (ID/IG) was used to assess the degree of graphitization. As shown in Figure 10, HPU-3 has the lowest ID/IG value, whereas pure PC/ABS has the largest ID/IG value. After UiO-66 is added to the sample HPU-2, its ID/IG value becomes higher than that of HP-7. These results suggest that HPU-3 has the highest degree of graphitization in the char layer, and the UiO-66 enhances char formation during combustion.

**Figure 11:**
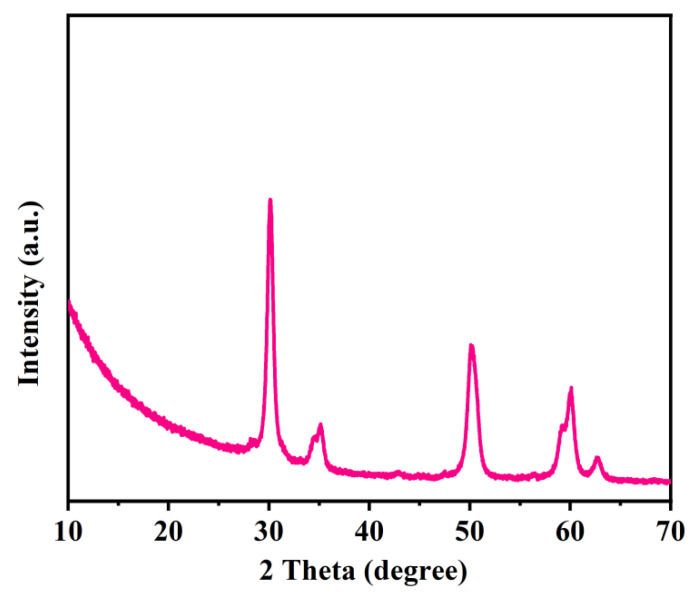
XRD patterns of UiO-66 after thermal degradation.

XRD was used to analyze the crystalline structure of the pyrolysis products of UiO-66. The prepared UiO-66 was placed in a muffle furnace at 600 °C for 0.5 h and collected for testing. As shown in Figure 11, the XRD pattern shows the characteristic peaks of the thermal barrier material, zirconium oxide. When the material burns, zirconium oxide can slow down the heat transfer rate and prevent the combustion products from spreading from the PC/ABS matrix to the burning area, to some extent. Therefore, after the material underwent intense combustion, although a large amount of the framework structure of UiO-66 was destroyed, its pyrolysis product—zirconium oxide—could still participate in smoke suppression.

**Figure 12:**
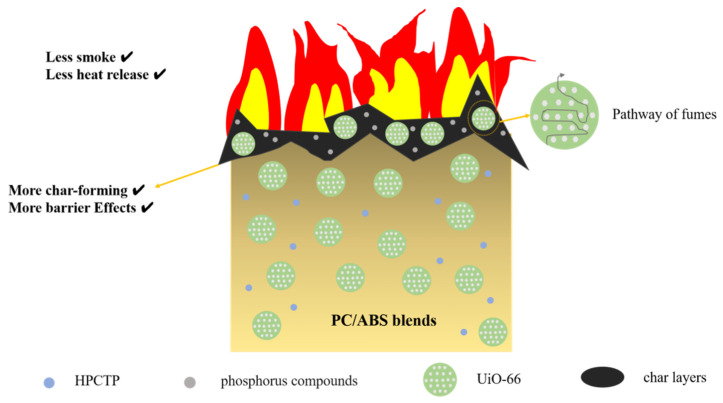
Illustration for PC/ABS blends’ flame-retardant mode of action.

Based on the above analysis, Figure 12 proposes the flame-retardant mode of action for UiO-66 in the PC/ABS blends. Generally, under constant heating from an external heat source, thermal degradation occurs at the first stage. Oxygen at the surface of the degrading polymer greatly promotes the thermo-oxidative process. The degrading polymer thus continuously produces volatile combustibles, which catch fire when they reach a certain concentration and temperature. A portion of the heat released by combustion further feeds the degrading polymer. In this work, by melt blending, UiO-66 and HPCTP can be dispersed homogenously into the PC/ABS blends. During the burning process, the incorporation of UiO-66 contributes to the formation of a continuous char layer, which acts as a protective shield for the underlying materials. One reason for this is that the metal oxides produced by UiO-66 during combustion expose metal sites that can positively participate in and catalyze the dehydrogenation or cross-linking reactions of PC/ABS pyrolysis into char. Additionally, UiO-66 is conducive to form a dense, stable graphitized char layer since the terephthalic acid ligand can enhance the degree of graphitization of the char layer^12^. As discussed above, this char layer can prevent heat radiation and reduce the release of volatile combustibles. Moreover, the porosity of UiO-66 remains stable in the early and middle stages of combustion, and it requires PC/ABS pyrolysis volatiles to overcome the tunneling effect. In the later stage of pyrolysis, UiO-66 partially decomposes into zirconium oxide, which can slow down the heat transfer rate as a thermal barrier material, and it prevents combustion products escaping from the PC/ABS matrix to the combustion zone. Therefore, in the PC/ABS/HPCTP blends, UiO-66 shows a cooperative effect on the flame-retardant performance of intumescent flame-retardant materials.

## 4. Conclusions

In this work, a zirconium-based metal-organic framework material (UiO-66) was synthesized to cooperatively enhance the flame retardancy of PC/ABS intumescent materials constructed with HPCTP via melt blending and two-screw extrusion. The successful preparation of UiO-66 was confirmed using FT-IR, XRD, XPS, and SEM analyses. Results indicate that UiO-66 has good thermal stability and a delaying effect on the pyrolysis of the intumescent flame-retardant materials. UiO-66 can significantly enhance the efficiency of HPCTP’s intumescent flame retardants. The flame-retardant PC/ABS blends with both UiO-66 and HPCTP show good flame retardancy. By compounding 7.0 wt% HPCTP with 3 wt% UiO-66, the blend shows LOI values of 27.0%, reaches a V-0 rating, and significantly improves resistance to combustion dripping. The CCT showed that UiO-66 suppresses heat release and smoke release in these materials. Finally, the flame-retardant mode of action in the blends is proposed, and UiO-66 has a cooperative effect on the flame-retardant performance of PC/ABS/HPCTP intumescent flame-retardant materials. This work provides a basis for the further development of flame-retardant PC/ABS.

## Figures and Tables

**Table 1:** Composition of flame-retardant PC/ABS blends.

Sample	PC/ABS (wt%)	HPCTP (wt%)	UiO-66 (wt%)
PC/ABS	70:30	0	0
HP-3.5	70:30	3.5	0
HP-7.0	70:30	7.0	0
HP-10.5	70:30	10.5	0
U-1.5	70:30	0	1.5
U-3.0	70:30	0	3
U-4.5	70:30	0	4.5
HPU-1	70:30	7.0	1
HPU-2	70:30	7.0	2
HPU-3	70:30	7.0	3

**Table 2:** LOI and vertical burning test results of PC/ABS blends.

Sample Name	HPCTP (wt%)	UiO-66 (wt%)	LOI (%)	UL-94	*t*_1_/s	*t*_2_/s	Dripping
PC/ABS	0	0	21.2	NR	>60	>60	YES
HP-3.5	3.5	0	22.7	NR	>60	>60	YES
HP-7.0	7.0	0	24.0	NR	50.2	>60	YES
HP-10.5	10.5	0	25.3	V-2	18.4	26.7	YES
U-1.5	0	1.5	23.2	NR	>60	>60	YES
U-3.0	0	3	23.7	NR	50.8	>60	YES
U-4.5	0	4.5	24.1	NR	39.6	45.1	NO
HPU-1	7.0	1	23.8	NR	39.2	>60	YES
HPU-2	7.0	2	25.2	V-2	17.2	28.8	YES
HPU-3	7.0	3	27.0	V-0	9.4	17.2	NO

## Data Availability

Data are contained within this article.

## Supplementary Materials

The following supporting information can be downloaded at: https://www.mdpi.com/article/10.3390/polym16142083/s1, Figure S1: Element distribution of UiO-66; Figure S2: 3D and 2D structures of UiO-66; Figure S3: tensile strain at break and tensile strength of PC/ABS and HPU-1, 2, 3; Table S1:Thermogravimetric data of UiO-66 and HPCTP; Table S2: Thermogravimetric data of PC/ABS composites; Table S3: PC/ABS composite cone calorimetry test data.
