# Supplementary material for: Development of a Zr-Based Metal-Organic Framework (UiO-66) for a Cooperative Flame Retardant in the PC/ABS
Source: Polymers (Basel). 2024 Jul 21;16(14):2083. doi: 10.3390/polym16142083 (PMC11281045; doi:10.3390/polym16142083)
Supplement: Supplementary file 1 [file polymers-16-02083-s001.zip › polymers-3092017-supplementary.pdf]

polymers

MDPI

Development of Zr-based metal-organic framework (UiO-66) for cooperative flame retardant in the PC/ABS

Shaojun Chen[a], Zerui Chen[a], Weifeng Bi[a], Wei Du[a], Ling Lin[a], Dasong Hu[a], Haitao Zhuo[b]*

Supplementary materials

Table S1. Thermogravimetric data of UiO-66 and HPCTP

	$T_{-5\%}$(°C)	$T_{-50\%}$(°C)	T_{MAX1}(°C)	T_{MAX2}(°C)	Residuesat 800°C(wt%)
UiO-66	106.9	549.6	144.8	548.9	33.92
HPCTP	319.5	376.3	392.3	—	0

Table S2. Thermogravimetric data of PC/ABS composites

	$T_{-5\%}$ (°C)	$T_{-50\%}$ (°C)	Maxlossrate (%/°C)	Residuesat 500°C(wt%)	Residuesat 800°C(wt%)
PC/ABS	409.5	454.5	1.70	13.4	9.0
U-1.5	401.8	452.5	1.69	15.2	9.1
U-3.0	405.6	457.4	1.42	18.9	12.2
U-4.5	392.9	445.9	1.57	20.1	14.0
HP-3.5	386.8	450.2	1.27	14.5	9.3
HP-7.0	386.7	456.1	1.18	17.2	11.3
HP-10.5	378.8	456.0	1.03	17.8	13.1
HPU-1	383.3	452.6	1.19	16.3	10.7
HPU-2	397.5	454.8	1.38	19.4	14.6
HPU-3	402.8	460.5	0.89	26.1	16.4

Table S3. PC/ABS composite cone calorimetry test data

Parameters	PC/ABS	HP-7	HPU-2	HPU-3
PHRR(kW/m^2)	221.9	188.4	169.9	149.3
t-PHPR (s)	127	152	147	152
TRR(kW/m^2)	25870	24230	20936	17315
TSR (m^2/m^2)	13.58	11.74	12.74	8.24
FGI	1.75	1.24	1.15	0.98

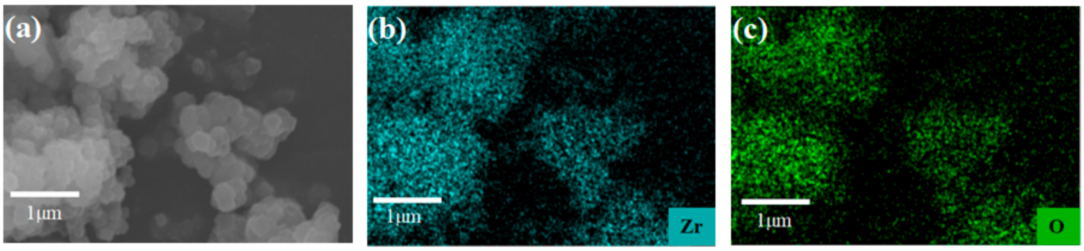

Figure S1. Element distribution of UiO-66

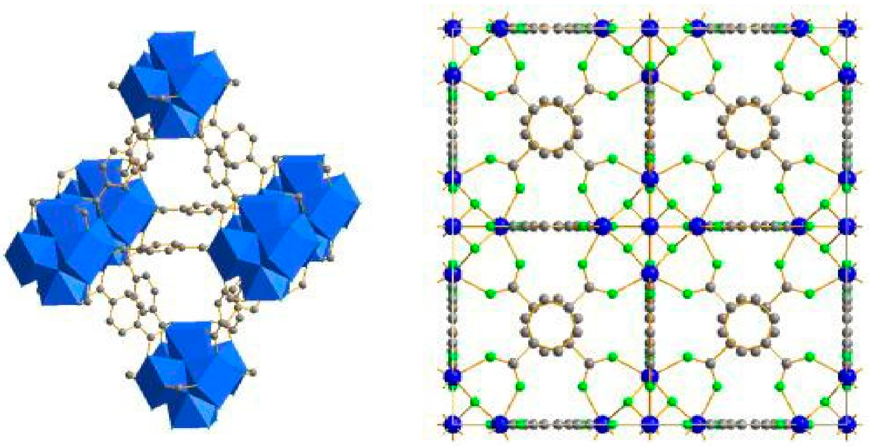

Figure S2. 3D and 2D structures of UiO-66

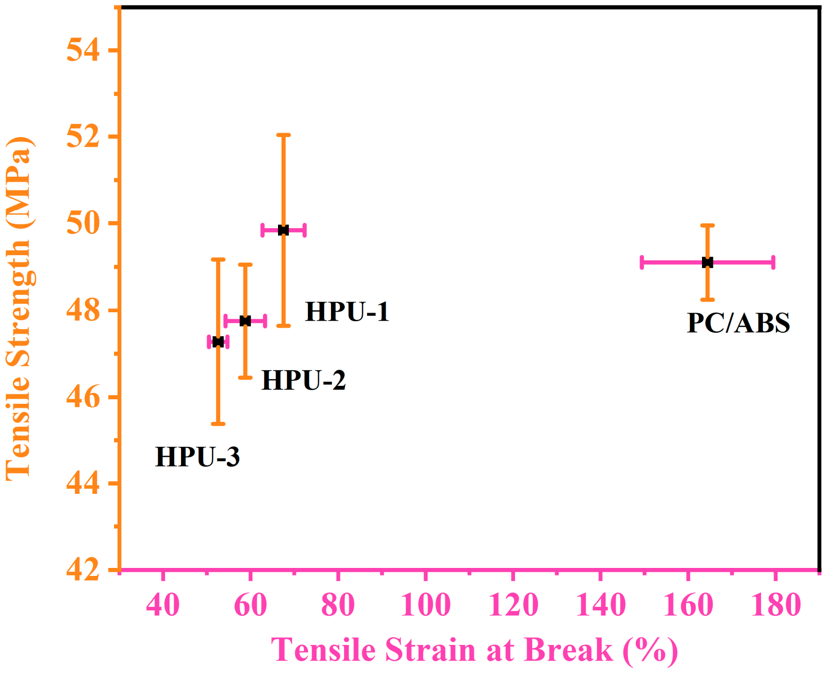

Figure S3. tensile strain at break and tensile strength of PC/ABS and HPU-1，2，3
